# Robust Anionic Framework Based on Sodium–Cerium Terephthalate

**DOI:** 10.3390/molecules30214195

**Published:** 2025-10-27

**Authors:** Nikita Nikandrov, Sofya Spasskaya, Marina Tedeeva, Alexander Kustov, Dmitry Tsymbarenko

**Affiliations:** 1 Faculty of Materials Science, Lomonosov Moscow State University, 119991 Moscow, Russia; 2 Department of Chemistry, Lomonosov Moscow State University, 119991 Moscow, Russia; 3 N. D. Zelinsky Institute of Organic Chemistry, Russian Academy of Sciences, 119991 Moscow, Russia

**Keywords:** metal–organic framework, cerium, X-ray structure, thermal behavior, pair distribution function, carbon monoxide oxidation

## Abstract

Synthesis of anionic metal–organic framework Na[Ce(BDC)_2_(DMF)_2_] based on cerium (III)–sodium terephthalate was performed. The crystal structure, studied by the Rietveld method, consists of anionic [Ce(BDC)_2_]^−^ layers, connected by interlayer sodium cations in a 3D network. Variable-temperature PXRD, total X-ray scattering with pair distribution function analysis, and DFT calculations revealed framework structure stability upon DMF elimination and thermal treatment up to 300 °C. Modification with copper cations was performed using wetness impregnation with a Cu(NO_3_)_2_ methanol solution to obtain a catalyst for carbon monoxide oxidation. Cu^2+^@Na[Ce(BDC)_2_(DMF)_2_] in situ decomposition leads to the catalytic activity of the resulting CuO/CeO_2_ composite during CO gas oxidation by air.

## 1. Introduction

Metal–organic frameworks (MOFs) are a class of compounds consisting of inorganic nodes (ions or polynuclear clusters), typically linked by di- or polytopic organic linkers. MOFs have attracted researchers’ attention due to their high porosity, providing a wide range of possible applications: gas storage [1] and separation [2], drug delivery [3], magnetism [4], luminescent thermometers [5], sensors [6], catalysis [7], etc.

In catalysis, cerium MOFs are being intensively researched because of the well-known ability of cerium to switch between the +3 and +4 oxidation states and thus be a redox catalyst [8,9,10]. Ce^4+^ MOFs usually consist of [Ce_6_O_4_(OH)_4_]^12+^ nodes, connected by organic linkers [11]. The most-studied example is Ce-UiO-66, belonging to the UiO-66 structure type first discovered for zirconium, which consists of such nodes connected with terephthalic acid (H_2_BDC) [12]. It was shown that the mixture of Ce-UiO-66 and TEMPO is an effective catalyst for benzyl alcohol to benzaldehyde oxidation [13].

MOFs containing Ce^3+^ exhibit vast structural diversity due to more miscellaneous coordination numbers (6–12). In most cases, Ce^3+^ MOFs consist of 1D chains [14,15,16], connected in three dimensions by di- or polytopic ligands [17]. Inorganic building units (IBUs) are mono- [18] or polynuclear [19,20,21] clusters with cerium ions coordinated by carboxylate groups, oxo/hydroxo bridging oxygen atoms and/or solvent molecules.

However, in aerobic oxidation reactions, for example, CO oxidation to CO_2_, MOFs do not demonstrate exceptional catalytic activity [22]. In order to catalyze such reactions, nanoparticles of catalytically active metals or their oxides are usually deposited on the surface of MOFs [23,24,25]. Another widely utilized approach involves obtaining MOF-derived catalysts [26,27]. Such catalysts demonstrate higher specific surface areas, compared with nanodispersed CeO_2_ obtained by rapid precipitation or hydrothermal methods, and feature higher catalytic activity in contrast with the original MOF.

Although in most cases a framework is electrically neutral, there are examples of so-called anionic MOFs which consist of a negatively charged 3D framework and cations in the framework’s voids to compensate for the charge [28,29,30]. Recent studies have shown growing interest in using anionic MOFs in potassium ion capacitors [31], solid-state electrolytes for lithium-ion batteries [32], proton conductors [33], etc.

For catalytic applications, anionic MOFs are of particular interest due to the possibility of conducting ion exchange between cations in MOF voids and cations of a future catalyst’s active phase [34].

Anionic MOFs are often obtained serendipitously as a result of dimethylformamide decomposition with dimethylamine formation, templating the construction of the metal–organic framework. Thus, scientific data about the functional properties of anionic frameworks are scarce. Previously, we reported on tailored synthesis of a cerium anionic metal–organic framework [(CH_3_)_2_NH_2_]_2_[Ce_2_(BDC)_4_(DMF)]·2H_2_O [35]. The formation of this MOF is templated by dimethylamine present in the reaction mixture. Thermal decomposition of the MOF results in nanodispersed CeO_2_ formation with S_BET_ = 182 m^2^/g.

In contrast, this work reports the ambient-condition synthesis of an anionic, layered sodium–cerium MOF. By replacing organic cations with interlayer sodium ions, we achieve enhanced structural robustness, a property which is typically absent in such frameworks. Further modification with copper cations allowed a CO oxidation catalyst to be obtained.

## 2. Results and Discussion

### 2.1. Synthesis

Metal–organic frameworks are usually synthesized under hydro- or solvothermal conditions due to necessity of overcoming kinetic factors during crystallization of coordination polymers with polytopic ligands. Such methods may lead to obtaining single crystals, significantly easing structure refinement. On the other hand, conducting the synthetic procedure in beakers is more convenient, scalable and reproducible. To obtain a high-crystalline product, slow ligand exchange must be ensured. Another obstacle is low scalability—it is challenging to synthesize large product quantities, needed for catalytic experiments. Our work proposes using a Ce_4_(OH)_2_(piv)_10_(H_2_O)_2_ polynuclear coordination polymer, which demonstrates low solubility in DMF, allowing slow exchange between pivalic and terephthalic acid in a beaker, with the desired product precipitating within several hours with high yield and product quantities.

Anionic MOFs are often obtained as a result of DMF decomposition accompanied by the formation of (CH_3_)_2_NH, which intercalates into the MOF voids and templates the framework topology. Serendipitous precipitation of several Na[La(BDC)_2_(DMF)_2_] crystals was previously achieved by mixing LaCl_3_ with H_2_BDC in the presence of NaOH [36]. In contrast to La^3+^, Ce^3+^ tends to be easily oxidized to Ce^4+^ in the presence of bases, which often leads to the precipitation of CeO_2_. In our case, an attempt to synthesize the target compound using NaOH as both the sodium source and base and CeCl_3_·7H_2_O as the cerium source resulted in the precipitation of a mixture of Na[Ce(BDC)_2_(DMF)_2_] and nanocrystalline CeO_2_ with an average grain size of 6.0 nm (Figure S1). Sodium hydroxide promotes hydrolysis of cerium salt used as a precursor and hinders the precipitation of the desired product.

Both methods 1 and 2 lead to the precipitation of the same product, Na[Ce(BDC)_2_(DMF)_2_]. It is essential to add a solution of Ce_4_(OH)_2_(piv)_10_(H_2_O)_2_ to a solution of H_2_BDC. Na[Ce(BDC)_2_(DMF)_2_] is a sodium–cerium terephthalate; therefore, synthesis in the presence of excess cerium leads to the precipitation of Ce_2_(BDC)_3_ with a one-dimensional chain structure. To obtain the desired product, an excess of H_2_BDC must be provided, which is achieved by adding the inorganic precursors into the solution of terephthalic acid.

The addition of (CH_3_)_2_NH provides binding of NO_3_^−^ anions resulting in [(CH_3_)_2_NH_2_]NO_3_ and Na_2_BDC formation. The presence of (CH_3_)_2_NH in the reaction mixture leads to precipitation of [(CH_3_)_2_NH_2_]_2_[Ce_2_(BDC)_4_(DMF)_2_], reported previously [35]. Thus, an excess of sodium salt is needed to promote the formation of the desired product.

### 2.2. X-Ray Crystal Structure

**Figure 1:**
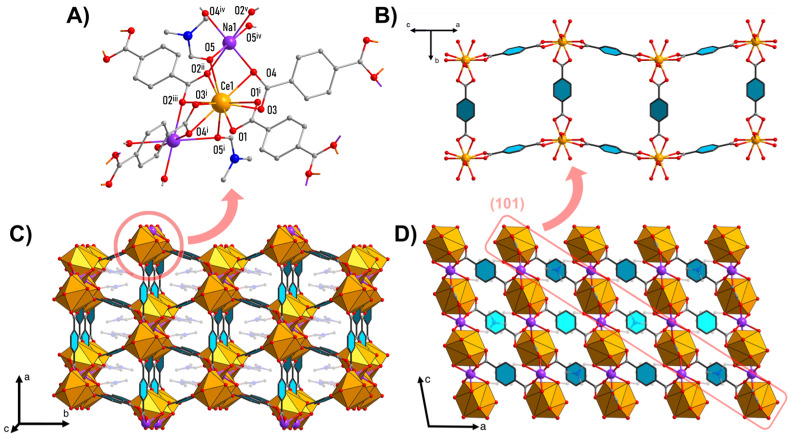
Crystal structure of Na[Ce(BDC)_2_(DMF)_2_]: cerium and sodium coordination environment (**A**); isolated layer structure (sodium atoms and DMF molecules are omitted for clarity) (**B**); packing diagram showing channels along *c* axis (**C**); packing diagram along *b* axis, showing layers’ arrangement in crystal (**D**). Color code: orange—[CeO_10_]; purple—Na; gray—C; red—O. DMF molecules are semi-transparent, hydrogen atoms are omitted for clarity. Symmetry codes: i—1 − x, y, 1.5 − z; ii—1 − x, −1 + y, 1.5 − z; iii—x, −1 + y, z; iv—1 − x, −y, 1 − z; v—x, 1 − y, −0.5 + z.

Na[Ce(BDC)_2_(DMF)_2_] crystallizes in the *C2/c* space group. The crystal structure contains one symmetrically independent Ce1 atom located on a twofold axis and one Na1 atom situated on an inversion center (Figure 1A). Each Ce1 is coordinated by the oxygen atoms of one chelating group (O1–C–O1^i^) and three chelate-bridging carboxylate groups (κ_1_:μ_3_-O2^ii^–C–O2^iii^, κ_1_:μ_2_-O3–C–O4, κ_1_:μ_2_-O3^i^–C–O4^i^) from BDC^2−^ linkers, as well as by two oxygen atoms (μ_2_-O5, μ_2_-O5^i^) from bridging DMF molecules (Table 1). The coordination environment of Ce1 with a coordination number (CN) of 10 is best described as a distorted tetradecahedron according to the Continuous Shape Measure (Table S1). The Na1 atom, situated in a distorted octahedral environment, is coordinated by six bridging oxygen atoms from four BDC^2−^ linkers (O4, O2^ii^, O4^iv^, O1^v^) and two DMF molecules (O5, O5^iv^).

Individual Ce1 ions are connected by four BDC^2−^ linkers into [[Ce(BDC)_2_]^−^]_∞_ layers parallel to the (101) plane, forming a distorted square network with cavity apertures of 9.0 Å × 6.8 Å (Figure 1B). The cavities are occupied by DMF molecules coordinated by Ce atoms from adjacent layers (Figure 1C). The Na^+^ ions are located in the interlayer space and, through bridging coordination with the BDC^2−^ ligands and DMF molecules, connect the layers into a three-dimensional framework (Figure 1D).

Secondary building units consist of 1D chains of [CeO_10_] and [NaO_6_] alternating polyhedrons with shared triangular faces along the c axis. One-dimensional chains are connected by BDC^2−^ linkers in three dimensions, forming a metal–organic framework.

### 2.3. Periodic DFT Calculations

To shed light on the structural stability of the framework upon removal of DMF molecules and to identify the corresponding structural changes, a series of periodic DFT calculations was performed. The XRD-based initial structural models of Na[Ce(BDC)_2_(DMF)_2_] and Na[Ce(BDC)_2_] with artificially removed DMF molecules were used for atomic position optimization. The lattice parameters were not varied, since PXRD data indicated that DMF removal practically does not lead to a change in the unit-cell dimensions.

Analysis of the resulting geometry of Na[Ce(BDC)_2_(DMF)_2_] reveals that the DFT model agrees well with the XRD data: The respective Ce–O distances differ by approximately 0.04 Å. The most significant discrepancies correspond to the weakest Na–O ionic interactions (Table 1). The calculated bond energy values (E_bond_) for the Ce–O contacts range from 40 to 60 kJ·mol^−1^, and the strongest bonds Ce–O1 are formed with only a chelate carboxyl group. The bond energy for the Ce–DMF contact (Ce–O5) is comparable to those for the Ce–O bonds involving the BDC^2−^ ligands. Sodium cations show much weaker interactions with bridging ligands. The total energies of the Ce–O and Na–O coordination bonds are 490.6 kJ·mol^−1^ and 142.6 kJ·mol^−1^, respectively.

The DFT model of Na[Ce(BDC)_2_] demonstrates that the removal of DMF molecules induces structural relaxation. The Ce1–O1, Ce1–O3, and Ce1–O4 bonds become shorter by ca. 0.06 Å, while Ce1–O2 remains unchanged, and Na1–O2 shortens instead by 0.11 Å (Table 1). It is worth noting that shortening of the bonds leads to their noticeable strengthening. As a result, the total energies of the coordination bonds Ce–O and Na–O after the DMF elimination, 475.2 and 133.8 kJ·mol^−1^, respectively, are very close to those in the initial Na[Ce(BDC)_2_(DMF)_2_] structure.

Thus, despite the significant contribution of DMF to the formation of the framework, its removal is easily compensated for by the relaxation of the framework and has little effect on the total energy of the coordination bonds.

### 2.4. Thermal Behavior

**Figure 2:**
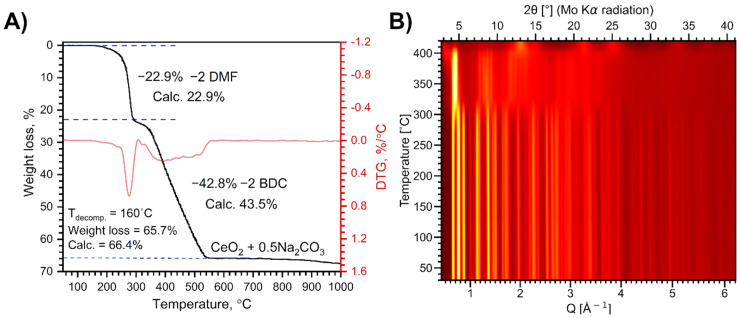
Weight loss curve of Na[Ce(BDC)_2_(DMF)_2_] thermal decomposition in air (**A**) and VT-PXRD patterns during the same process (**B**).

Thermal decomposition of Na[Ce(BDC)_2_(DMF)_2_] in air proceeds in two main stages (Figure 2A). The first stage of weight loss, occurring at 190–295 °C, corresponds to the elimination of two coordinated solvent molecules. The experimental weight loss of 22.9% is in perfect agreement with the calculated value of 22.9%. During the second stage, the resulting Na[Ce(BDC)_2_] undergoes multistep combustion at 320–540 °C, leading to the formation of CeO_2_ and Na_2_CO_3_. The total weight loss of 65.7% at 700 °C agrees well with the calculated value of 66.4%. A further weight loss observed at 900–1000 °C is attributed to the evaporation of Na_2_CO_3_.

VT-PXRD analysis reveals two phase transformations of the MOF upon heating in air up to 420 °C (Figure 2B). The XRD pattern of the MOF sample remains unchanged upon heating to 310 °C (Figure S2) and coincides with that of the as-synthesized Na[Ce(BDC)_2_(DMF)_2_], despite the elimination of DMF molecules according to the TGA data. Thus, the loss of coordinated DMF molecules does not lead to structural collapse. The refined lattice parameters show a gradual increase with rising temperature due to thermal expansion, while the growth of the *a* parameter slows above 160 °C as DMF molecules continue to depart (Figure S3).

At 310 °C, a structural reorganization occurs, resulting in the formation of a [Ce_2_(BDC)_3_] phase previously observed [35] upon heating the related anionic MOF [(CH_3_)_2_NH_2_]_2_[Ce_2_(BDC)_4_(DMF)_2_] to 350 °C. This phase remains stable up to 380 °C and decomposes upon further heating, forming nanocrystalline CeO_2_.

**Figure 3:**
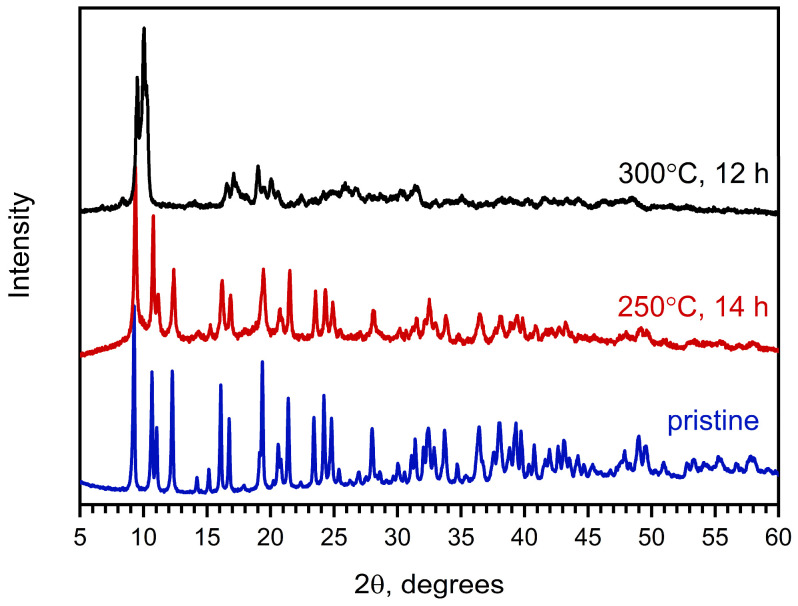
PXRD patterns of as-obtained Na[Ce(BDC)_2_(DMF)_2_] (**blue**) and products of its activation at 250 °C for 14 h under dynamic vacuum (**red**) and at 300 °C under dynamic vacuum for 12 h (**black**).

Moreover, ex situ experiments involving heating of Na[Ce(BDC)_2_(DMF)_2_] powder under dynamic vacuum at 250 and 300 °C for 12–14 h confirm the VT-PXRD results (Figure 3). Specifically, the PXRD pattern of the residue obtained after treatment at 300 °C differs from that of the as-synthesized MOF. The pattern of the Na[Ce(BDC)_2_] sample obtained after treatment at 250 °C is similar to that of Na[Ce(BDC)_2_(DMF)_2_], although the peaks become broader and less intense, indicating a reduction in crystallinity. The elimination of DMF at this stage was confirmed by CHN analysis and gravimetry. The Rietveld-refined unit-cell parameters of Na[Ce(BDC)_2_] are as follows: *a* = 16.918(4) Å, *b* = 11.694(3) Å, *c* = 12.705(3) Å, β = 100.876(17)°. The observed changes can be considered negligible compared with those of the as-synthesized sample (Δa = +0.004 Å, Δb = −0.005 Å, Δc = +0.007 Å, Δβ = −0.05°), indicating the rigidity of the framework.

**Figure 4:**
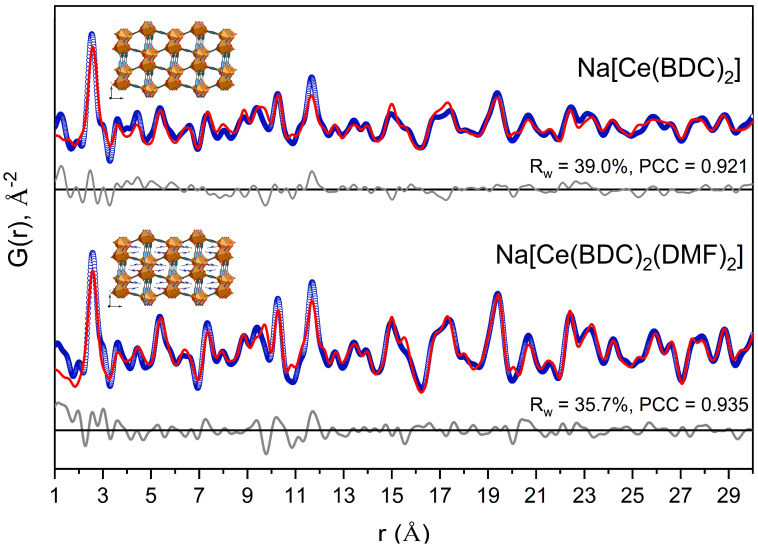
Experimental PDF data of as-synthesized Na[Ce(BDC)_2_(DMF)_2_] and Na[Ce(BDC)_2_] (**blue**), and their fits using corresponding periodic models (**red**). Difference curves (**gray**) are offset for clarity. PCC stands for Pearson correlation coefficient.

Total X-ray scattering combined with pair distribution function (PDF) analysis was performed to examine changes in the local structure upon DMF elimination (Figure 4). The results indicate both local and long-range structure retention after DMF elimination with hardly noticeable interatomic distance changes. The Pearson correlation coefficient (PCC) between the PDFs of the as-synthesized sample and the same sample after DMF elimination is 0.934. After treatment at 250 °C for 14 h under dynamic vacuum, the PDF shows a noticeable damping relative to that of the as-synthesized sample, reflecting a reduction in structural order caused by DMF departure.

The PDF of the as-synthesized Na[Ce(BDC)_2_(DMF)_2_] sample is well described by the corresponding structural model (PCC = 0.935), whereas the PDF of the heat-treated sample is best fitted using the Na[Ce(BDC)_2_] structural model with DMF molecules artificially removed (PCC = 0.921). It is worth noting that fitting the second PDF with the Na[Ce(BDC)_2_(DMF)_2_] structural model yields a lower correlation between the experimental and calculated data (PCC = 0.905) (Figure S4), confirming the elimination of DMF upon heating.

### 2.5. Specific Surface Area and Pore Size Distribution

**Figure 5:**
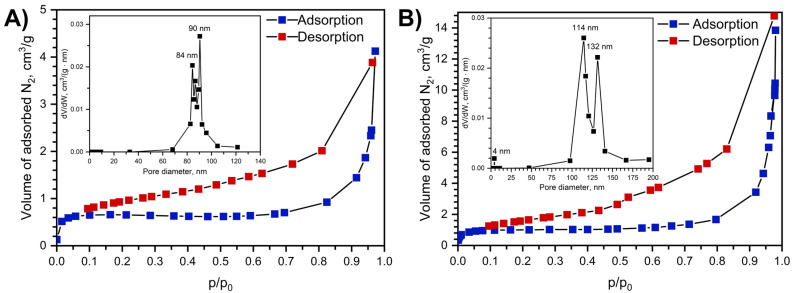
Nitrogen adsorption isotherms at 77K before (**A**) and after sample treatment at 250 °C for 14 h (**B**) and adsorption–desorption isotherms and pore size distribution, calculated with BJH model using desorption isotherm branch shown as insets.

The obtained adsorption–desorption isotherms (Figure 5) are Type-III (IUPAC classification), corresponding to nonporous materials. An increase in nitrogen uptake in the p/p_0_ < 0.1 region indicates the presence of micropores with diameters less than 2 nm. The specific surface areas, calculated using the BET model, were found to be 1 m^2^/g for Na[Ce(BDC)_2_(DMF)_2_] and 2 m^2^/g for Na[Ce(BDC)_2_].

Crystallographic porosity and specific surface area after the artificial removal of guest molecules were calculated using the Zeo++ package [37] with probe radii of 1.2 Å and 1.77 Å. For a probe radius of 1.2 Å, the micropore volume is 0.0837 cm^3^·g^−1^ and the specific surface area is 1656.07 m^2^·g^−1^, which falls within the typical range for MOFs. When the probe radius is increased to 1.77 Å, corresponding to the radius of an N_2_ molecule, both the micropore volume and the specific surface area drop to zero. This behavior is attributed to cavities that are isolated from each other by narrow channels. The high rigidity of the metal–organic framework prevents nitrogen molecules from entering the cavities.

### 2.6. CO Oxidation

**Figure 6:**
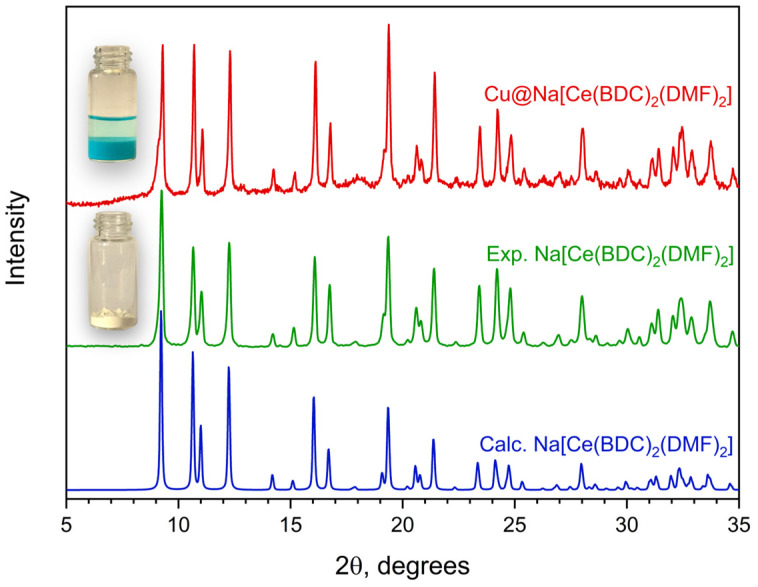
PXRD patterns of Na[Ce(BDC)_2_(DMF)_2_] before (**green**) and after copper impregnation (**red**). (**Blue**) line represents calculated PXRD pattern for corresponding structure.

To obtain an effective catalyst for a heterogeneous redox process, it is reasonable to combine several chemical elements with variable oxidation states. Therefore, the cerium-based material was modified by impregnating Na[Ce(BDC)_2_(DMF)_2_] with a methanolic Cu(NO_3_)_2_ solution. The absorption of copper from the solution by the MOF is evidenced by the formation of a blue-colored precipitate. EDX analysis (Figure S5b) of the resulting Cu@Na[Ce(BDC)_2_(DMF)_2_] sample shows a relatively low copper content of 3.3 wt%. According to PXRD data (Figure 6), the MOF sample retains its structural integrity and crystallinity after modification. Moderate changes in the relative intensity of several peaks can be attributed to the incorporation of Cu^2+^ ions into the framework and the partial substitution of DMF by CH_3_OH molecules.

**Figure 7:**
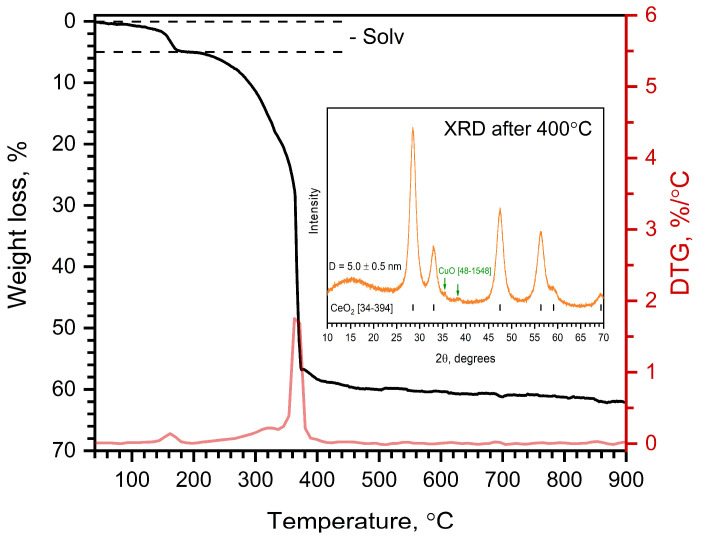
TGA curve of Cu@Na[Ce(BDC)_2_(DMF)_2_] in air. XRD pattern of sample heated under same conditions up to 400 °C is shown as inset.

Figure 7 illustrates the TGA and DTG curves of Cu@Na[Ce(BDC)_2_(DMF)_2_]. Impregnation of the metal–organic framework with a methanolic Cu(NO_3_)_2_ solution leads to the substitution of DMF by MeOH molecules and results in pronounced changes in the thermal behavior. The DTG curve indicates two consecutive processes: the elimination of solvent molecules and the decomposition of the organic linker. The observed weight loss in the first stage is 4.9%, which corresponds to the removal of one MeOH molecule. The second stage of weight loss consists of two overlapping substages that occur consecutively. These stages correspond to the decomposition of the organic residues and the formation of CuO/CeO_2_ and Na_2_CO_3_. The XRD pattern of the sample heated to 400 °C shows the presence of two phases: CeO_2_ nanoparticles with an average grain size of 5 nm and CuO. The Na_2_CO_3_ phase was not detected because of its low scattering intensity compared with CeO_2_. The temperature of total decomposition decreases from 540 °C to 419 °C compared with the pristine Na[Ce(BDC)_2_(DMF)_2_]. This can be attributed to the presence of the CuO phase, which promotes low-temperature oxidation of the organic molecules.

**Figure 8:**
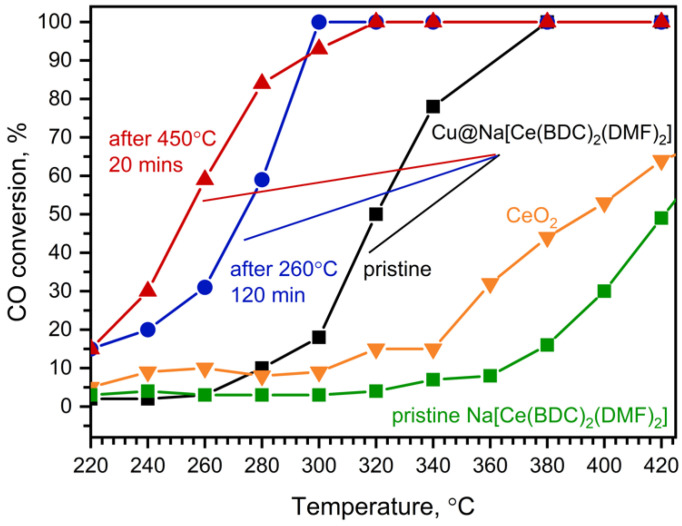
CO conversion on Cu@Na[Ce(BDC)_2_(DMF)_2_]: pristine (**black**), after treatment in CO+O_2_ gas flow at 260 °C for 2 h (**blue**), after calcination in CO+O_2_ gas flow at 450 °C for 20 min (**red**), and on CeO_2_ (**orange**) and pristine Na[Ce(BDC)_2_(DMF)_2_] (**green**).

The fresh Cu@Na[Ce(BDC)_2_(DMF)_2_] catalyst exhibits modest activity in the CO oxidation reaction up to 340 °C, at which point MOF decomposition occurs, resulting in the formation of CeO_2_ on the catalyst surface and immediate increase in the reaction rate (Figure 8, black). The maximum reaction rate of the fresh Cu@Na[Ce(BDC)_2_(DMF)_2_] catalyst was 347.1 mmol CO g^−1^ cat h^−1^, and 100% CO conversion was reached at 380 °C.

The catalyst underwent one subsequent test under the same conditions, which resulted in an increase in catalytic activity (Figure 8, blue): T_100_ decreased from 380 °C to 300 °C. The observed improvement in oxidative activity is attributed to MOF decomposition. The PXRD pattern of the catalyst treated at 260 °C for 2 h under the experimental conditions (Figure S6b) shows the presence of the Na[Ce(BDC)_2_(DMF)_2_] phase along with quartz. We assume that copper oxide is the active phase in this temperature range, promoting both CO and MOF oxidation with the formation of CeO_2_. After thermal decomposition, the catalyst composition was calculated to be 6 wt% CuO/CeO_2_.

Treatment of the fresh catalyst at 450 °C for 20 min resulted in a further increase in catalytic activity (Figure 8, red). Although T_100_ is higher than that of the catalyst treated at 260 °C for 2 h (T_100_ = 320 °C vs. 300 °C), 50% conversion was achieved at approximately 250 °C, which is about 20 °C lower. This may be attributed to the activation of copper species on the catalyst surface, which results in increased low-temperature oxidative activity. On the other hand, the increase in T_100_ might be due to CeO_2_ sintering, leading to a decrease in both surface area and high-temperature catalytic activity. The activated sample demonstrates stable performance for at least 20 h (Figure S7). All investigated Cu@Na[Ce(BDC)_2_(DMF)_2_] samples exhibit higher catalytic activity in CO oxidation than commercially available CeO_2_.

Table 2 summarizes the catalytic activity data for different metal–organic frameworks in the CO oxidation reaction. Although HKUST-1 [38] demonstrates superior catalytic performance in CO oxidation, among the listed cerium-based MOFs, Cu@Na[Ce(BDC)_2_(DMF)_2_] exhibits the lowest T_50_ value, presumably due to copper–cerium interactions that lead to the generation of catalytically active sites [39,40].

CeO_2_ nanoparticles are known to possess oxygen vacancies; thus, the surface of the CuO–CeO_2_ interface presumably consists of Ce^3+^–V_o_–Cu^+^ active sites. The role of copper is to adsorb CO molecules on the surface, forming Cu^+^–CO species. The diffusion of lattice oxygen from CeO_2_ to the interface leads to formation of Ce^4+^–O^2−^–Cu^2+^–CO. Finally, CO is oxidized by lattice oxygen, CO_2_ is released, and the cerium and copper ions are reduced to +3 and +1, respectively. The resulting oxygen vacancies are then replenished by gaseous oxygen [41]. According to previous reports [42], CuO/CeO_2_ catalysts exhibit higher catalytic activity in CO oxidation than CuO–CeO_2_ solid solutions. Thus, the formation of a well-defined CuO–CeO_2_ interface is crucial for achieving high oxidative activity [43].

**Table 2:** Catalytic activities of different cerium metal–organic frameworks in CO oxidation reaction.

System	Temperature of 50% CO Conversion (T_50_), °C	Activation Procedure	Reference
HKUST-1	235	In reaction atmosphere during the first catalytic run	[38]
Ce-BTC	310	None	[44]
Cu@Na[Ce(BDC)_2_(DMF)_2_]	253	450 °C for 20 min in reaction atmosphere	This work
MIL-53(Ce)	275	None	[45]

After the CO oxidation performance test was completed, no traces of the MOF phase were detected in the catalyst. The PXRD pattern (Figure S6c) contains only reflections corresponding to quartz and CeO_2_. Because the scattering power of Na_2_CO_3_ is much lower than that of CeO_2_ and quartz, no diffraction peaks from this phase were observed.

## 3. Materials and Methods

Cerium nitrate, pivalic acid (2,2-dimethylpropionic acid, Hpiv), and terephthalic acid (1,4-benzenedicarboxylic acid, H_2_BDC) were purchased from Sigma-Aldrich (Darmstadt, Germany). N,N-dimethylformamide (DMF), sodium nitrate, copper nitrate, and dimethylamine (33% aqueous solution) were obtained from a local supplier (Rushim, Moscow, Russia). All reagents were of analytical grade and were used as received without further purification. The Ce_4_(OH)_2_(piv)_10_(H_2_O)_2_ powder was synthesized and characterized following the method described previously by our group [46].

The C, H, and N contents were measured using a PerkinElmer 2400 CHNS/O Organic Elemental Analyzer (PerkinElmer, Waltham, MA, USA); the Ce content was calculated from the weight residue of the TG-DTA analysis in air at 1000 °C. TG-DTA data were collected in an air atmosphere using a Derivatograph Q-1500 D (MOM, Budapest, Hungary) (heating rate: 10 °C·min^−1^; sample mass: 50 mg). FT-IR spectra were recorded on a PerkinElmer Spectrum 3 FTIR spectrometer (Perkin Elmer, Waltham, MA, USA) in the attenuated total reflectance (ATR) geometry in the wavenumber range of 550–4000 cm^−1^. Powder XRD data for phase analysis were collected on a Rigaku Miniflex 600 diffractometer (Rigaku, Tokyo, Japan) (600 W, CuKα radiation, λ = 1.54187 Å) equipped with D/tex Ultra 1D detector with Kβ filter.

Total X-ray scattering data for PDF analysis of Na[Ce(BDC)_2_(DMF)_2_] and Na[Ce(BDC)_2_] samples, placed in 0.5 mm Kapton capillaries, were collected on a Bruker D8 QUEST single-crystal X-ray diffractometer (Bruker AXS, Karlsruhe, Germany) equipped with a microfocus Incoatec IμS 3.0 Mo tube (λ = 0.71073 Å) and CMOS Photon III detector in the Q-range of 0.35–16.97 Å^−1^ [47]. The raw series of 2D frames were processed using the FormagiX 0.9.9b software [48] and transformed into PDFs with PDFgetX3 [49] employing a Q-range of 0.3–14.0 Å^−1^. Refinements were carried out with DiffPy-CMI 3.0.1 [50] using the crystal structure model of Na[Ce(BDC)_2_(DMF)_2_] and derived model of Na[Ce(BDC)_2_], obtained by artificial exclusion of DMF molecules from Na[Ce(BDC)_2_(DMF)_2_].

Textural characteristics were determined based on the nitrogen adsorption isotherms measured at 77K using an IMC ProSurf-V 1220 analyzer (IMC-systems, Moscow, Russia). Before the measurements, the samples were activated at 250 °C and 5 × 10^−2^ mmHg for 2 h to remove physically adsorbed solvent molecules. The specific surface area was calculated according to the Brunauer–Emmett–Teller (BET) method, and the pore size distribution was derived from the desorption branch of the isotherm using Barrett–Joyner–Halenda (BJH) analysis. Additionally, sample activation was performed in dynamic conditions upon heating at 250 °C in nitrogen flow for 2 h using Sorbi-MS analyzer (Meta, Novosibirsk, Russia), and the following nitrogen adsorption experiment confirmed the same value of the specific surface area.

### 3.1. Synthesis of Na[Ce(BDC)_2_(DMF)_2_]

*Method 1.* Powders of H_2_BDC (1.478 g, 8.9 mmol), NaNO_3_ (0.3831 g, 4.5 mmol) and Ce_4_(OH)_2_(piv)_10_(H_2_O)_2_ (1.838 g, 1.3 mmol) were dispersed in 130 mL of DMF. Then, 3.7 mL of a 30% aqueous dimethylamine solution was added under stirring. The resulting solution was placed into a 250 mL Teflon-lined stainless-steel autoclave, sealed, and heated at 120 °C for 48 h. The white precipitate was filtered off, washed three times with 2 mL portions of DMF, and left to dry in air. The yield was 75%.

*Method 2.* Powders of Ce_4_(OH)_2_(piv)_10_(H_2_O)_2_ (1.838 g, 1.3 mmol), NaNO_3_ (0.3831 g, 4.5 mmol) and H_2_BDC (1.478 g, 8.9 mmol) were dissolved in 130 mL of DMF. The resulting clear solution was stirred in a glass beaker at room temperature for 2 h until a white-yellowish crystalline precipitate was obtained. The precipitate was filtered off and thoroughly washed several times with DMF, then dried in air. The yield was 55%.

Anal. Calc. for C_22_H_22_CeN_2_NaO_10_ (MW 637.53, wt%): 41.45 C; 3.48 H; 4.39 N; 21.98 Ce. Found: 40.82 C; 3.55 H; 4.72 N, 21.4 Ce. FT-IR (ATR, ν, cm^−1^): 670m, 747s, 763s, 821w, 834vs, 866w, 887w, 981w, 1003vw, 1016w, 1063w, 1097w, 1115m, 1149w, 1249w, 1309w, 1390vs, 1403vs, 1438m, 1505m, 1544m, 1571s, 1594m, 1650s, 2895vw, 2944vw, 2975vw. PXRD: *C2/c*, a = 16.9144(10) Å, b = 11.6992(6) Å, c = 12.6978(9) Å, β = 100.928(5)°.

### 3.2. Preparation of Na[Ce(BDC)_2_]

A sample of pristine Na[Ce(BDC)_2_(DMF)_2_] was heated at 250 °C under low pressure (5 × 10^−2^ mmHg) for 14 h to remove coordinated DMF molecules.

Anal. Calc. for C_16_H_8_CeNaO_8_ (MW 490.92, wt %): 39.11 C; 1.64 H; 28.52 Ce. Found: 39.21 C; 1.81 H; 26.9 Ce. FT-IR (ATR, ν, cm^−1^): 670m, 747s, 763s, 821m, 833vs, 865w, 887w, 981w, 1005vw, 1017w, 1062w, 1098w, 1115m, 1152w, 1249w, 1310w, 1389vs, 1401vs, 1438m, 1506m, 1544s, 1568s, 1594m, 1651s, 2897vw, 2943vw, 2975vw. PXRD: *C2/c*, a = 16.918(4) Å, b = 11.694(3) Å, c = 12.705(3) Å, β = 100.876 (17)°.

### 3.3. Modification of Na[Ce(BDC)_2_(DMF)_2_] with Copper Ions

A powder sample of Na[Ce(BDC)_2_(DMF)_2_] (2.0 g, 3.1 mmol) was dispersed in 200 mL of a methanolic solution of Cu(NO_3_)_2_·3H_2_O (0.1038 g, 0.43 mmol). The resulting mixture was then stirred for 24 h at room temperature. The precipitate was filtered out and left undisturbed in air.

### 3.4. Catalytic Performance in Carbon Monoxide Oxidation

CO oxidation experiments were conducted in a CO + air flow (1:10 V/V) within the temperature range of 25–420 °C. The volume ratio of CO:O_2_ was 1:2. To prevent sample spilling during the experiment due to MOF oxidation and decrease in sample volume, the catalyst was mixed with α-quartz at a 1:1 (m/m) ratio.

Catalytic properties of formed MOF-derived CeO_2_ samples were studied in CO oxidation reactions at atmospheric pressure in the temperature range 25–420 °C in a flow catalytic setup with a steel reactor with an inside diameter of 4 mm. The reaction mixture CO + air was fed into the reactor with a CO/air ratio of 1:10 V/V, and the total flow rate of the gas mixture was 77 mL/min. To prevent sample spilling during the experiment due to MOF oxidation and a decrease in sample volume, the catalyst was mixed with α-quartz at a 2:3 (m/m) ratio. The catalysts were used without preliminary activation if not stated otherwise.

Cu@Na[Ce(BDC)_2_(DMF)_2_] or pristine Na[Ce(BDC)_2_(DMF)_2_] sample loading was 200 mg and 300 mg of quartz sand (grain size 0.25–0.5 mm). MOF-derived CeO_2_ was produced by preliminary heating of MOF within the catalytic setup at 450 °C for 20 min. On-line analysis of the reaction products was carried out using a Chromatec-Crystal 5000 gas chromatograph (Chromatec, Yoshkar-Ola, Russia) equipped with a thermal conductivity detector, SS316 3 m × 2 mm columns, Hayesep Q 80/100 mesh, and CaA molecular sieves. The temperature of the column was ramped according to the following program: 40 °C for 1.5 min, increasing to 100 °C at a rate of 15 °C/min. The quantitative analysis was carried out by the absolute calibration method.

### 3.5. X-Ray Crystallography

Powder XRD data of Na[Ce(BDC)_2_(DMF)_2_] for structure solution and Rietveld refinement were collected on a Tongda TD-3700 diffractometer (Dandong Tongda Science & Technology, Dandong City, China) operated in symmetrical reflection geometry (1200 W, CuKα radiation, λ = 1.54187 Å, Dectris Mythen2 R 1D detector with Kβ filter). The powder sample was placed in a glass sample holder and continuously spun around its normal during measurements to minimize preferred orientation and improve counting statistics.

**Figure 9:**
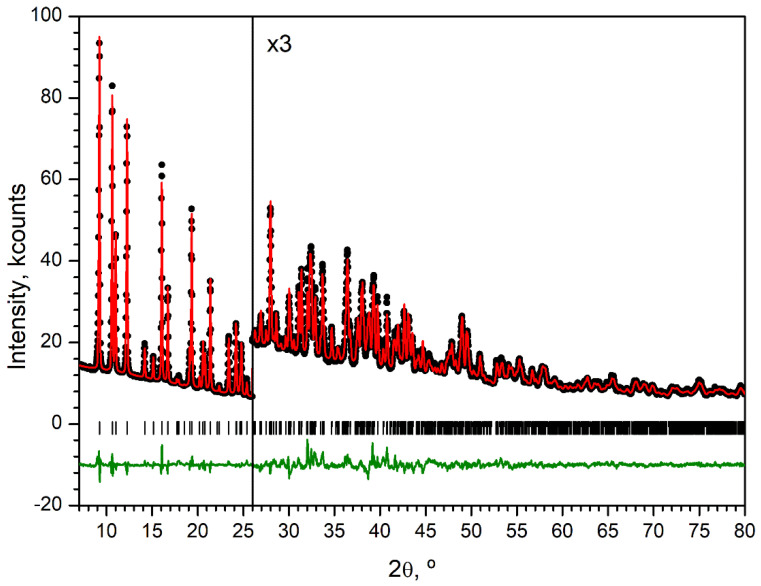
PXRD pattern (λ = 1.5419 Å) of Ce-MOF at 293K (**black circles**), Rietveld refinement fit (**red solid line**), difference profile (offset **green line**), and positions of Bragg peaks (**vertical bars**).

The XRD pattern (Figure 9) was indexed in the monoclinic crystal system with F_30_ = 117 (|Δ2θ|= 0.006°, 45 theoretical peaks) by the dichotomy method as implemented in DICVOL91 [51]. Full-profile unit-cell refinement, peak-intensity extraction using the le Bail method, and final structure refinement using the Rietveld method were performed in JANA2006 [52]. The positions of Ce, Na and O atoms were solved using the Superflip method [53]. Other atoms were found from difference Fourier syntheses. The structure was refined with soft restraints on C–C, C–O, and C–N bond lengths and the corresponding valence angles. Four isotropic atomic displacement parameters were used—for Ce, Na, non-hydrogen atoms of BDC^2−^ and non-hydrogen atoms of DMF. Hydrogen atoms were placed in idealized positions and treated in a riding mode. The pattern was fitted with an eleven-term Chebyshev polynomial background and a five-term pseudo-Voigt shape function with asymmetry corrected by axial divergence. The details of the data collection and refinement parameters are summarized in Table 3. Figure 9 shows the refined powder XRD profile. CCDC 2,461,298 contains crystallographic information associated with this paper.

### 3.6. DFT Calculations

DFT calculations with periodic boundary conditions were performed using the projector-augmented wave (PAW) method implemented in the VASP 5.4.4 software package using the PBE functional and the Gamma-centered Brillouin zone [54,55,56,57]. The energy cutoff of the plane wave in all calculations was set to 450 eV. The Grimme D3 dispersion correction was applied [58]. Geometry optimization was carried out using data obtained from a single-crystal X-ray experiment as an initial model. The electron density distribution function calculated in VASP was analyzed in terms of the QT-AIM theory [59] using the AIM-UC v.1.6.10 software package [60]. The values of the energy of coordination bonds E_Bond_ were estimated using the Espinosa–Molins–Lecomte correlation as ½*v*(r_CP_), where *v*(r_CP_) is the potential energy density at the bond critical point [61].

## 4. Conclusions

An approach to the synthesis of anionic metal–organic frameworks through the interaction of a metal salt with an acidic linker in the presence of additional cation species has been successfully applied to obtain a new cerium(III)-based metal–organic framework. The crystal structure of the obtained Na[Ce(BDC)_2_(DMF)_2_] compound consists of anionic [Ce(BDC)_2_]^−^ layers with sodium ions in the interlayer space, forming a 3D network with DMF molecules occupying cavities.

According to TGA, VT-PXRD, and PDF data, the MOF releases DMF molecules upon thermal activation at 250 °C but retains its crystal structure up to 300 °C with minor changes in unit-cell parameters due to thermal expansion. PDF analysis of pristine and activated MOF samples confirms the structural rigidity after elimination of DMF molecules.

Periodic DFT calculations reveal that the coordination bonds between Ce ions and the BDC^2−^ anions and DMF molecules possess comparable bonding energies. Despite this fact, elimination of DMF leads to enforcement of remaining coordination bonds; thus, the overall energy of Ce–O interactions virtually does not change, preventing structure collapse.

Modification of the Na[Ce(BDC)_2_(DMF)_2_] metal–organic framework by copper ions resulted in the formation of Cu@Na[Ce(BDC)_2_(DMF)_2_] catalyst with 3.3 wt% copper content. The catalyst was tested in a heterogeneous redox process of carbon monoxide oxidation by air. To achieve maximum catalytic activity, preliminary activation of the catalyst is needed to ensure total MOF conversion to a CuO/CeO_2_ composite. The latter demonstrates long-term catalytic stability.

## Figures and Tables

**Table 1:** Selected interatomic distances and bond energies in Na[Ce(BDC)_2_(DMF)_2_] and Na[Ce(BDC)_2_] according to XRD data and DFT calculations. Symmetry codes: i—1 − x, y, 1.5 − z; ii—1 − x, −1 + y, 1.5 − z; iii—x, −1 + y, z; iv—1 − x, −y, 1 − z; v—x, 1 − y, −0.5 + z.

	Na[Ce(BDC)_2_(DMF)_2_]			Na[Ce(BDC)_2_]	
Contact	XRD Distance, Å	DFT		DFT	
		Distance, Å	E_Bond_ kJ/mol	Distance, Å	E_Bond_ kJ/mol
Ce1–O1, Ce1–O1^i^	2.47(3)	2.547	59.3	**2.479**	**74.6**
Ce1–O2^ii^, Ce1–O2^iii^	2.67(3)	2.647	43.6	2.657	43.1
Ce1–O3, Ce1–O3^i^	2.55(3)	2.593	51.5	**2.537**	**62.9**
Ce1–O4, Ce1–O4^i^	2.61(3)	2.622	46.7	**2.564**	**57.0**
Ce1–O5, Ce1–O5^i^	2.62(3)	2.614	44.2		—
Na1–O2^ii^, Na1–O2^v^	2.231(14)	2.383	20.3	**2.270**	**31.4**
Na1–O4, Na1–O4^iv^	2.23(4)	2.219	35.5	2.222	35.5
Na1–O5, Na1–O5^iv^	2.54(4)	2.458	15.5		—

The most noticeable changes are indicated in bold.

**Table 3:** Crystallographic and refinement data for Na[Ce(BDC)_2_(DMF)_2_].

	Na[Ce(BDC)_2_(DMF)_2_]
Formula	C_22_H_22_CeN_2_NaO_10_
Formula weight (g·mol^−1^)	637.53
Diffractometer	Tongda TD-3700
Wavelength (Å)	1.54187 (Cu Kα)
Data collection method	θ-θ scan
Temperature (K)	293
Crystal system	Monoclinic
Space group	*C2/c*
*a* (Å)	16.9144(10)
*b* (Å)	11.6992(6)
*c* (Å)	12.6978(9)
β (°)	100.928(5)
V (Å^3^)	2467.1(3)
Z	4
Color, habit	White, powder
Sample dimensions (mm)	30 × 30 × 0.5
D_calc_ (g·cm^−3^)	1.7164
μ (mm^−1^)	14.952
2θ range (º)	7–80
Observed reflections	769
Parameters, restrains	71, 57
R_Bragg_, R_p_, ωR_p_	0.0338, 0.0269, 0.0360
Goodness-of-fit ^1^	3.09
Absorption correction	not required
ρ_min_, ρ_max_ (eÅ^−3^)	−0.74, 0.65

^1^ Goodness-of-fit is calculated on the overall powder pattern intensities.

## Data Availability

The primary data presented in this study are available on request from the corresponding author.

## Supplementary Materials

The following supporting information can be downloaded at https://www.mdpi.com/article/10.3390/molecules30214195/s1. Figure S1. PXRD pattern of product, obtained by using CeCl_3_ and NaOH to synthesize Na[Ce(BDC)_2_(DMF)_2_]. Figure S2. PXRD patterns, obtained in situ during heating the Na[Ce(BDC)_2_(DMF)_2_] sample, and Pearson correlation coefficients between adjacent VT-PXRD patterns. Figure S3. Temperature dependence of experimental unit-cell parameter obtained by Rietveld refinement of VT-PXRD data. Figure S4. PDFs of as-obtained Na[Ce(BDC)_2_(DMF)_2_] and the same sample after DMF elimination in dynamic vacuum at 250 °C. Figure S5. SEM picture of Cu@Na[Ce(BDC)_2_(DMF)_2_] and overview EDX spectrum. Figure S6. PXRD patterns of Na[Ce(BDC)_2_(DMF)_2_] after impregnation with Cu^2+^ and after CO oxidation experiments. Figure S7. Results of catalytic experiments; Table S1: Continuous Shape Measures.

## References

[1] Alezi D., Belmabkhout Y., Suyetin M., Bhatt P.M., Weselinski L.J., Solovyeva V., Adil K., Spanopoulos I., Trikalitis P.N., Emwas A.H. (2015). MOF Crystal Chemistry Paving the Way to Gas Storage Needs: Aluminum-Based soc-MOF for CH_4_, O_2_, and CO_2_ Storage. J. Am. Chem. Soc..

[2] Qian Q., Asinger P.A., Lee M.J., Han G., Rodriguez K.M., Lin S., Benedetti F.M., Wu A.X., Chi W.S., Smith Z.P. (2020). MOF-Based Membranes for Gas Separations. Chem. Rev..

[3] Cao J., Li X., Tian H. (2020). Metal-Organic Framework (MOF)-Based Drug Delivery. Curr. Med. Chem..

[4] Zhao X., Liu S., Tang Z., Niu H., Cai Y., Meng W., Wu F., Giesy J.P. (2015). Synthesis of Magnetic Metal-Organic Framework (MOF) for Efficient Removal of Organic Dyes from Water. Sci. Rep..

[5] Zhao D., Rao X., Yu J., Cui Y., Yang Y., Qian G. (2015). Design and Synthesis of an MOF Thermometer with High Sensitivity in the Physiological Temperature Range. Inorg. Chem..

[6] Jo Y., Jo Y.K., Lee J., Jang H.W., Hwang I., Yoo D.J. (2023). MOF-Based Chemiresistive Gas Sensors: Toward New Functionalities. Adv. Mater..

[7] Doonan C.J., Sumby C.J. (2017). Metal–Organic Framework Catalysis. CrystEngComm.

[8] Machida M., Murata Y., Kishikawa K., Zhang D., Ikeue K. (2008). On the Reasons for High Activity of CeO_2_ Catalyst for Soot Oxidation. Chem. Mater..

[9] Montini T., Melchionna M., Monai M., Fornasiero P. (2016). Fundamentals and Catalytic Applications of CeO_2_-Based Materials. Chem. Rev..

[10] Thammachart M., Meeyoo V., Risksomboon T., Osuwan S. (2001). Catalytic Activity of CeO_2_–ZrO_2_ Mixed Oxide Catalysts Prepared via Sol–Gel Technique: CO Oxidation. Catal. Today.

[11] Jacobsen J., Wegner L., Reinsch H., Stock N. (2020). Ce-MIL-140: Expanding the Synthesis Routes for Cerium(IV) Metal–Organic Frameworks. Dalton Trans..

[12] Cavka J.H., Jakobsen S., Olsbye U., Guillou N., Lamberti C., Bordiga S., Lillerud K.P. (2008). A New Zirconium Inorganic Building Brick Forming Metal Organic Frameworks with Exceptional Stability. J. Am. Chem. Soc..

[13] Lammert M., Wharmby M.T., Smolders S., Bueken B., Lieb A., Lomachenko K.A., Vos D.D., Stock N. (2015). Cerium-Based Metal Organic Frameworks with UiO-66 Architecture: Synthesis, Properties and Redox Catalytic Activity. Chem. Commun..

[14] Weng S.-F., Wang Y.-H., Lee C.-S. (2012). New Metal-Organic Frameworks of [M(C_6_H_5_O_7_)(C_6_H_6_O_7_)(C_6_H_7_O_7_)(H_2_O)]⋅H_2_O (M=La, Ce) and [Ce_2_(C_2_O_4_)(C_6_H_6_O_7_)_2_]⋅4H_2_O. J. Solid State Chem..

[15] Atzori C., Lomachenko K.A., Øien-Ødegaard S., Lamberti C., Stock N., Barolo C., Bonino F. (2019). Disclosing the Properties of a New Ce(III)-Based MOF: Ce_2_(NDC)_3_(DMF)_2_. Cryst. Growth Des..

[16] Han Y.-F., Zhou X.-H., Zheng Y.-X., Shen Z., Song Y., You X.-Z. (2008). Syntheses, Structures, Photoluminescence, and Magnetic Properties of Nanoporous 3D Lanthanide Coordination Polymers with 4,4′-Biphenyldicarboxylate Ligand. CrystEngComm.

[17] Jacobsen J., Ienco A., D’Amato R., Costantino F., Stock N. (2020). The Chemistry of Ce-Based Metal–Organic Frameworks. Dalton Trans..

[18] Gil-Hernández B., Maclaren J.K., Höppe H.A., Pasán J., Sanchiz J., Janiak C. (2012). Homochiral Lanthanoid(III) Mesoxalate Metal–Organic Frameworks: Synthesis, Crystal Growth, Chirality, Magnetic and Luminescent Properties. CrystEngComm.

[19] Qi J.-L., Zheng Y.-Q., Xu W., Zhu H.-L., Lin J.-L., Chang H.-S. (2013). New Ce(Iii) Sulfate–Tartrate-Based MOFs: An Insight into the Controllable Self-Assembly of Acentric Metal–Organic Complexes. CrystEngComm.

[20] Han Y., Li X., Li L., Ma C., Shen Z., Song Y., You X. (2010). Structures and Properties of Porous Coordination Polymers Based on Lanthanide Carboxylate Building Units. Inorg. Chem..

[21] D’Arras L., Sassoye C., Rozes L., Sanchez C., Marrot J., Marre S., Aymonier C. (2014). Fast and Continuous Processing of a New Sub-Micronic Lanthanide-Based Metal–Organic Framework. New J. Chem..

[22] Lin A., Ibrahim A.A., Arab P., El-Kaderi H.M., El-Shall M.S. (2017). Palladium Nanoparticles Supported on Ce-Metal–Organic Framework for Efficient CO Oxidation and Low-Temperature CO_2_ Capture. ACS Appl. Mater. Interfaces.

[23] Xu J., Liu J., Li Z., Wang X., Wang Z. (2019). Synthesis, Structure and Properties of Pd@MOF-808. J. Mater. Sci..

[24] Jiang H.-L., Liu B., Akita T., Haruta M., Sakurai H., Xu Q. (2009). Au@ZIF-8: CO Oxidation over Gold Nanoparticles Deposited to Metal−Organic Framework. J. Am. Chem. Soc..

[25] Qian L., Zhen Z., Jian L., Yue-Chang W., Gui-Yuan J., Ai-Jun D. (2014). Pd Nanoparticles Deposited on Metal-Organic Framework of MIL-53(Al): An Active Catalyst for CO Oxidation. Acta Phys.-Chim. Sin..

[26] Bai C., Li A., Yao X., Liu H., Li Y. (2016). Efficient and Selective Aerobic Oxidation of Alcohols Catalysed by MOF-Derived Co Catalysts. Green Chem..

[27] Gong X., Wang W.-W., Fu X.-P., Wei S., Yu W.-Z., Liu B., Jia C.-J., Zhang J. (2018). Metal-Organic-Framework Derived Controllable Synthesis of Mesoporous Copper-Cerium Oxide Composite Catalysts for the Preferential Oxidation of Carbon Monoxide. Fuel.

[28] Al-Maythalony B.A., Shekhah O., Swaidan R., Belmabkhout Y., Pinnau I., Eddaoudi M. (2015). Quest for Anionic MOF Membranes: Continuous Sod-ZMOF Membrane with CO_2_ Adsorption-Driven Selectivity. J. Am. Chem. Soc..

[29] Li P., Vermeulen N.A., Gong X., Malliakas C.D., Stoddart J.F., Hupp J.T., Farha O.K. (2016). Design and Synthesis of a Water-Stable Anionic Uranium-Based Metal–Organic Framework (MOF) with Ultra Large Pores. Angew. Chem..

[30] Bhattacharyya S., Chakraborty A., Jayaramulu K., Hazra A., Maji T.K. (2014). A Bimodal Anionic MOF: Turn-off Sensing of Cu II and Specific Sensitization of Eu III. Chem. Commun..

[31] Aphirakaramwong C., Akintola O., Plass C.T., Sawangphruk M., Plass W., Balducci A. (2023). Improving the Performance of an Anionic MOF by Counter Cation Replacement as Electrode Material in a Full Cell Setup of a Potassium Ion Capacitor. RSC Adv..

[32] Hou T., Xu W. (2023). Deep Dive into Anionic Metal-Organic Frameworks Based Quasi-Solid-State Electrolytes. J. Energy Chem..

[33] Sun H.-X., Wang H.-N., Fu Y.-M., Meng X., He Y.-O., Yang R.-G., Zhou Z., Su Z.-M. (2022). A Multifunctional Anionic Metal–Organic Framework for High Proton Conductivity and Photoreduction of CO_2_ Induced by Cation Exchange. Dalton Trans..

[34] Chakraborty A., Bhattacharyya S., Hazra A., Ghosh A.C., Maji T.K. (2016). Post-Synthetic Metalation in an Anionic MOF for Efficient Catalytic Activity and Removal of Heavy Metal Ions from Aqueous Solution. Chem. Commun..

[35] Grebenyuk D., Shaulskaya M., Shevchenko A., Zobel M., Tedeeva M., Kustov A., Sadykov I., Tsymbarenko D. (2023). Tuning the Cerium-Based Metal–Organic Framework Formation by Template Effect and Precursor Selection. ACS Omega.

[36] Long L.-S., Hu J.-Y., Ren Y.-P., Sun Z.-G., Huang R.-B., Zheng L.-S. (2002). Crystal structure of a 3D coordination polymer: Sodium lanthanide terephthalate N, N-dimethylformamide solvate. Main Group Met. Chem..

[37] Willems T.F., Rycroft C.H., Kazi M., Meza J.C., Haranczyk M. (2012). Algorithms and Tools for High-Throughput Geometry-Based Analysis of Crystalline Porous Materials. Micropor. Mesopor. Mat..

[38] Zamaro J.M., Pérez N.C., Miró E.E., Casado C., Seoane B., Téllez C., Coronas J. (2012). HKUST-1 MOF: A Matrix to Synthesize CuO and CuO–CeO_2_ Nanoparticle Catalysts for CO Oxidation. Chem. Eng. J..

[39] Stawowy M., Jagódka P., Matus K., Samojeden B., Silvestre-Albero J., Trawczyński J., Łamacz A. (2020). HKUST-1-Supported Cerium Catalysts for CO Oxidation. Catalysts.

[40] Rojas-Buzo S., Salusso D., Le T.-H.T., Ortuño M.A., Lomachenko K.A., Bordiga S. (2024). Unveiling the Role and Stabilization Mechanism of Cu + into Defective Ce-MOF Clusters during CO Oxidation. J. Phys. Chem. Lett..

[41] Martínez-Arias A., Fernández-García M., Gálvez O., Coronado J.M., Anderson J.A., Conesa J.C., Soria J., Munuera G. (2000). Comparative Study on Redox Properties and Catalytic Behavior for CO Oxidation of CuO/CeO_2_ and CuO/ZrCeO_4_ Catalysts. J. Catal..

[42] Hossain S.T., Azeeva E., Zhang K., Zell E.T., Bernard D.T., Balaz S., Wang R. (2018). A Comparative Study of CO Oxidation over Cu-O-Ce Solid Solutions and CuO/CeO_2_ Nanorods Catalysts. Appl. Surf. Sci..

[43] Shang H., Zhang X., Xu J., Han Y. (2017). Effects of Preparation Methods on the Activity of CuO/CeO_2_ Catalysts for CO Oxidation. Front. Chem. Sci. Eng..

[44] Zhang X., Hou F., Li H., Yang Y., Wang Y., Liu N., Yang Y. (2018). A Strawsheave-like Metal Organic Framework Ce-BTC Derivative Containing High Specific Surface Area for Improving the Catalytic Activity of CO Oxidation Reaction. Micropor. Mesopor. Mat..

[45] Tan H.-Y., Zhou Y., Yan Y.-F., Wu D.-Y., Hu W.-B., Shi X.-Y. (2017). Metal Organic Framework Cu/MIL-53(Ce)-Mediated Synthesis of Highly Active and Stable CO Oxidation Catalysts. Inorg. Chem. Commun..

[46] Grebenyuk D., Zobel M., Polentarutti M., Ungur L., Kendin M., Zakharov K., Degtyarenko P., Vasiliev A., Tsymbarenko D. (2021). A Family of Lanthanide Hydroxo Carboxylates with 1D Polymeric Topology and Ln_4_ Butterfly Core Exhibits Switchable Supramolecular Arrangement. Inorg. Chem..

[47] Tsymbarenko D., Grebenyuk D., Burlakova M., Zobel M. (2022). Quick and Robust PDF Data Acquisition Using a Laboratory Single-Crystal X-Ray Diffractometer for Study of Polynuclear Lanthanide Complexes in Solid Form and in Solution. J. Appl. Crystallogr..

[48] Tsymbarenko D. (2025). FormagiX v.0.9.9b. 2D XRD Processing Software. https://formagix.org/..

[49] Juhás P., Davis T., Farrow C.L., Billinge S.J.L. (2013). *PDFgetX3*: A Rapid and Highly Automatable Program for Processing Powder Diffraction Data into Total Scattering Pair Distribution Functions. J. Appl. Crystallogr..

[50] Juhás P., Farrow C.L., Yang X., Knox K.R., Billinge S.J.L. (2015). Complex Modeling: A Strategy and Software Program for Combining Multiple Information Sources to Solve Ill Posed Structure and Nanostructure Inverse Problems. Acta Crystallogr. A Found. Adv..

[51] Boultif A., Louër D. (1991). Indexing of Powder Diffraction Patterns for Low-Symmetry Lattices by the Successive Dichotomy Method. J. Appl. Crystallogr..

[52] Petříček V., Dušek M., Palatinus L. (2014). Crystallographic Computing System JANA2006: General Features. Z. Kristallogr. Cryst. Mater..

[53] Palatinus L., Chapuis G. (2007). *SUPERFLIP*–A Computer Program for the Solution of Crystal Structures by Charge Flipping in Arbitrary Dimensions. J. Appl. Crystallogr..

[54] Kresse G., Furthmüller J. (1996). Efficiency of Ab-Initio Total Energy Calculations for Metals and Semiconductors Using a Plane-Wave Basis Set. Comput. Mater. Sci..

[55] Kresse G., Hafner J. (1994). Ab Initio Molecular-Dynamics Simulation of the Liquid-Metal–Amorphous-Semiconductor Transition in Germanium. Phys. Rev. B.

[56] Kresse G., Furthmüller J. (1996). Efficient Iterative Schemes for Ab Initio Total-Energy Calculations Using a Plane-Wave Basis Set. Phys. Rev. B.

[57] Kresse G., Hafner J. (1993). Ab Initio Molecular Dynamics for Liquid Metals. Phys. Rev. B.

[58] Grimme S., Antony J., Ehrlich S., Krieg H. (2010). A Consistent and Accurate *Ab Initio* Parametrization of Density Functional Dispersion Correction (DFT-D) for the 94 Elements H-Pu. J. Chem. Phys..

[59] Bader R.F.W. (1985). Atoms in Molecules. Acc. Chem. Res..

[60] Vega D., Almeida D. (2014). AIM-UC: An Application for QTAIM Analysis. J. Comput. Methods Sci. Eng..

[61] Espinosa E., Molins E., Lecomte C. (1998). Hydrogen Bond Strengths Revealed by Topological Analyses of Experimentally Observed Electron Densities. Chem. Phys. Lett..

