# Supplementary material for: Robust Anionic Framework Based on Sodium–Cerium Terephthalate
Source: Molecules. 2025 Oct 27;30(21):4195. doi: 10.3390/molecules30214195 (PMC12610006; doi:10.3390/molecules30214195)
Supplement: Supplementary file 1 [file molecules-30-04195-s001.zip › molecules-3904181-supplementary.pdf]

molecules

Article

Robust Anionic Framework Based on Sodium–Cerium Terephthalate

Nikita Nikandrov [1], Sofya Spasskaya [2], Marina Tedeeva [2], Alexander Kustov [2,3] and Dmitry Tsymbarenko [2,*]

[1] Faculty of Materials Science, Lomonosov Moscow State University, Moscow 119991, Russia;
[2] Department of Chemistry, Lomonosov Moscow State University, Moscow 119991, Russia;
[3] N. D. Zelinsky Institute of Organic Chemistry, Russian Academy of Sciences, Moscow 119991, Russia;
* Correspondence: tsymbarenko@gmail.com;

Abstract

Synthesis of anionic metal–organic framework $Na[Ce(BDC)_2(DMF)_2]$ based on cerium (III)–sodium terephthalate was performed. The crystal structure, studied by the Rietveld method, consists of anionic $[Ce(BDC)_2]^-$ layers, connected by interlayer sodium cations in a 3D network. Variable-temperature PXRD, total X-ray scattering with pair distribution function analysis, and DFT calculations revealed framework structure stability upon DMF elimination and thermal treatment up to 300 °C. Modification with copper cations was performed using wetness impregnation with a $Cu(NO_3)_2$ methanol solution to obtain a catalyst for carbon monoxide oxidation. Cu^{2+}@$Na[Ce(BDC)_2(DMF)_2]$ in situ decomposition leads to the catalytic activity of the resulting CuO/CeO_2 composite during CO gas oxidation by air.

Supplementary Materials

Table of contents

Figure S1. PXRD pattern of product, obtained by using $CeCl_3$ and NaOH to synthesize $Na[Ce(BDC)_2(DMF)_2]$.

Table S1. Continuous Shape Measures.

Figure S2. PXRD patterns, obtained *in situ* during heating the $Na[Ce(BDC)_2(DMF)_2]$ sample and Pearson correlation coefficients between adjacent VT-PXRD patterns.

Figure S3. Temperature dependence of experimental unit cell parameter obtained by Rietveld refinement of VT-PXRD data.

Figure S4. PDF's of as-obtained $Na[Ce(BDC)_2(DMF)_2]$ and the same sample after DMF elimination in dynamic vacuum at 250˚C.

Figure S5. SEM picture of $Cu@Na[Ce(BDC)_2(DMF)_2]$ and overview EDX spectrum.

Figure S6. PXRD patterns of $Na[Ce(BDC)_2(DMF)_2]$ after impregnation with Cu^{2+} and after CO oxidation experiments.

Figure S7. Results of catalytic experiments.

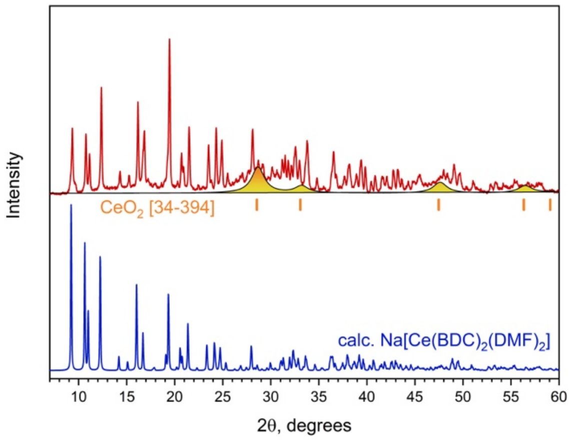

Figure S1. PXRD pattern of product, obtained by using $CeCl_3$ and NaOH to synthesize Na[Ce(BDC)$_2$(DMF)$_2$]. Calculated PXRD pattern for Na[Ce(BDC)$_2$(DMF)$_2$] **(blue)** and experimental PXRD pattern **(red)** of sample, synthesized following method described in literature [52] using $CeCl_3 \cdot 7H_2O$ as cerium source and NaOH as both sodium source and base. Orange-yellow area indicates peaks corresponding to nanocrystalline CeO_2 with grain size of 6.0 nm.

Table S1. Continuous Shape Measures.

[CeO_{10}]		
Polyhedron label	Polyhedron name	Continuous Shape Measures
DP-10	Decagon	33.589
EPY-10	Enneagonal pyramid	22.883
OBPY-10	Octagonal bipyramid	13.201
PPR-10	Pentagonal prism	11.937
PAPR-10	Pentagonal antiprism	8.797
JBCCU-10	Bicapped cube J15	5.990
JBCSAPR-10	Bicapped square antiprism J17	6.977
JMBIC-10	Metabidiminished icosahedron J62	4.103
JATDI-10	Augmented tridiminished icosahedron J64	17.828
JSPC-10	Sphenocorona J87	6.505
SDD-10	Staggered Dodecahedron (2:6:2)	3.320
**TD-10**	**Tetradecahedron (2:6:2)**	**2.471**
HD-10	Hexadecahedron (2:6:2) or (1:4:4:1)	3.189
[NaO_6]		
HP-6	Hexagon	25.728
PPY-6	Pentagonal pyramid	27.351
**OC-6**	**Octahedron**	**3.667**
TPR-6	Trigonal prism	14.978
JPPY-6	Johnson pentagonal pyramid	29.605

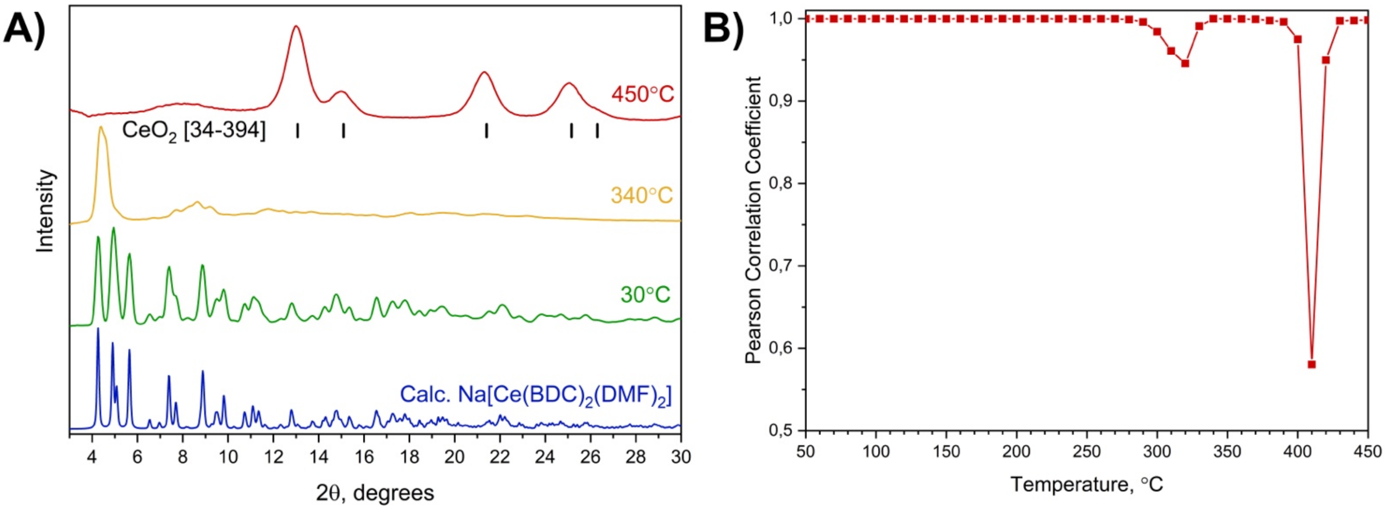

Figure S2. Mo Kα PXRD patterns, obtained *in situ* during heating the Na[Ce(BDC)$_2$(DMF)$_2$] sample **(A)** and Pearson correlation coefficients between adjacent VT-PXRD patterns **(B)**.

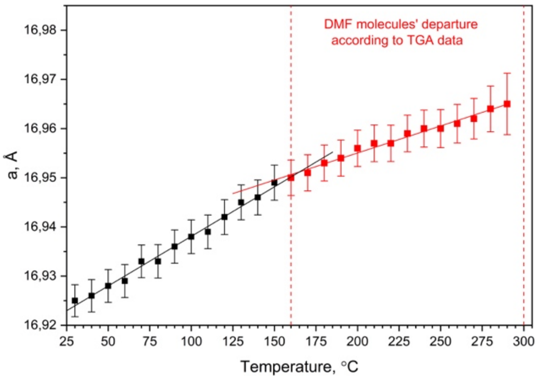

Figure S3. Temperature dependence of unit cell parameter *a* obtained by Rietveld refinement of experimental unit cell parameter *a* using VT-PXRD data. Black and red lines represent corresponding linear fits of parameter *a* temperature dependence.

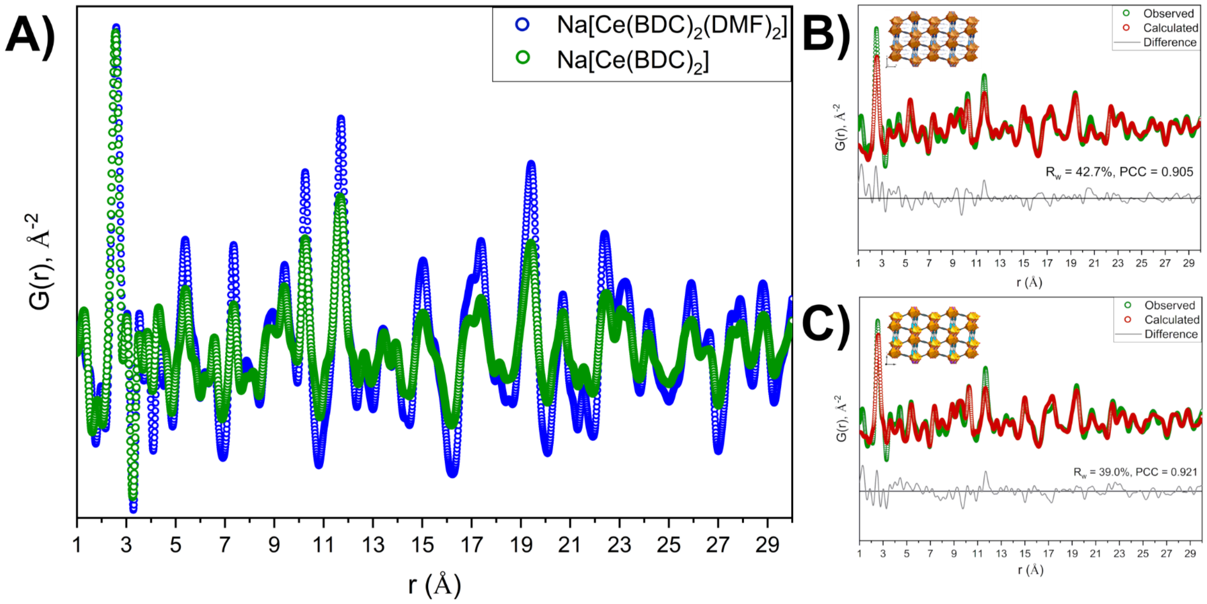

Figure S4. PDF's of as-obtained Na[Ce(BDC)$_2$(DMF)$_2$] and the same sample after DMF elimination in dynamic vacuum at 250°C for 14 hours (Na[Ce(BDC)$_2$]) **(A)**; Na[Ce(BDC)$_2$] PDF fitting using structure model of Na[Ce(BDC)$_2$(DMF)$_2$] **(B)**; Na[Ce(BDC)$_2$] PDF fitting using structure model of Na[Ce(BDC)$_2$(DMF)$_2$] with artificially extracted all DMF molecules **(C)**.

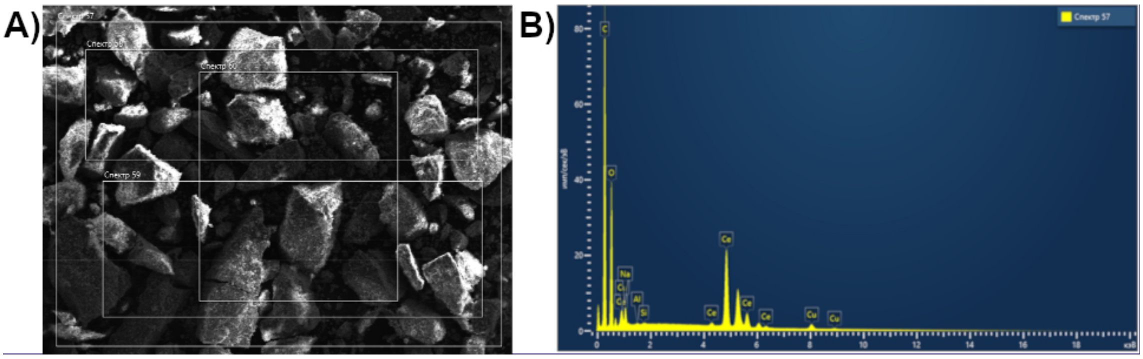

Figure S5. SEM picture of Cu@Na[Ce(BDC)$_2$(DMF)$_2$] **(A)** and overview EDX spectrum **(B)**.

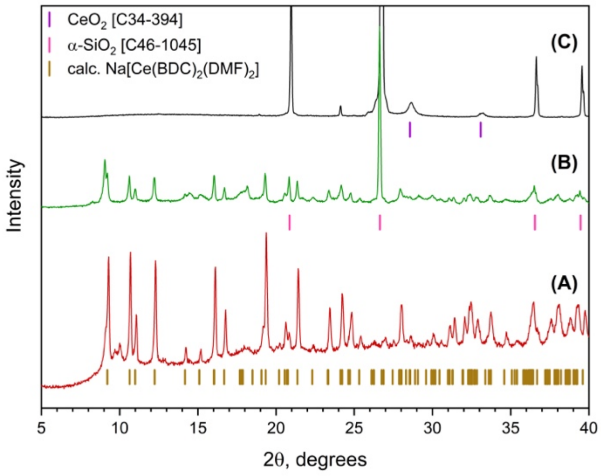

Figure S6. PXRD patterns of Na[Ce(BDC)$_2$(DMF)$_2$] after impregnation with Cu^{2+} **(A)**, catalyst after treatment at CO oxidation experiment conditions for 2 hours at 260˚C **(B)** and catalyst after CO oxidation performance test was completed **(C)**.

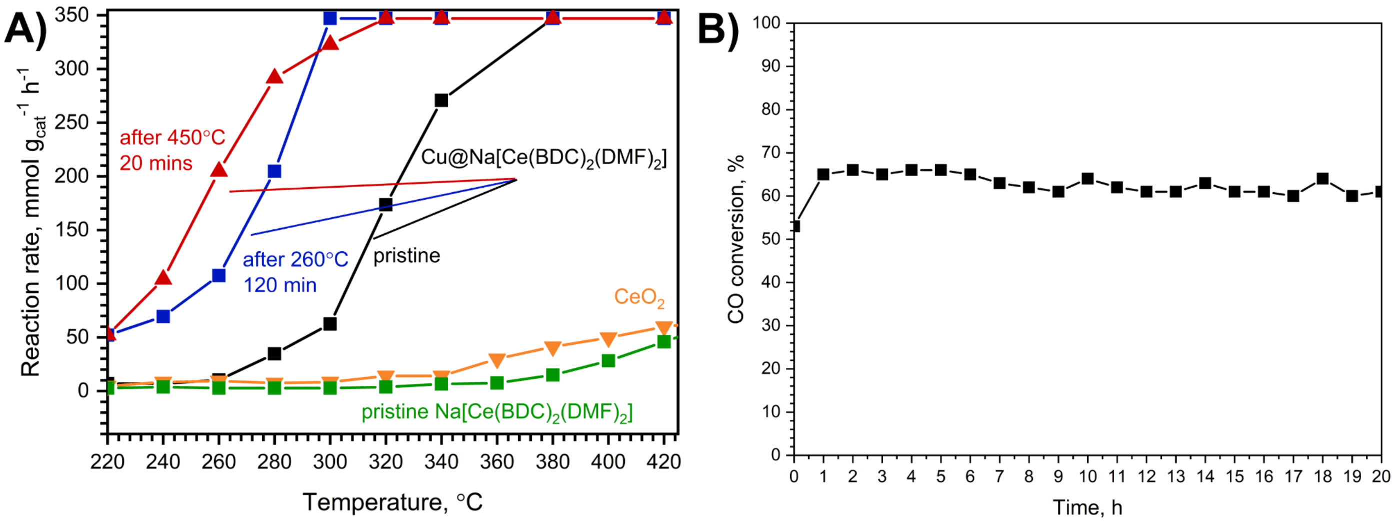

Figure S7. Results of catalytic experiments: **(A)** CO oxidation reaction rate on Cu@Na[Ce(BDC)$_2$(DMF)$_2$] — pristine **(black)**, after treatment in CO+O_2 gas flow at 260˚C for 2 hours **(blue)**, after calcination in CO+O_2 gas flow at 450˚C for 20 minutes **(red)**; on CeO_2 **(orange)** and pristine Na[Ce(BDC)$_2$(DMF)$_2$] **(green)**. **(B)** Catalyst derived from Cu@Na[Ce(BDC)$_2$(DMF)$_2$] stability measurement at T = 260˚C.
